# Existing practices, gaps and strategies to promote perinatal mental health in Ghana

**DOI:** 10.3389/fgwh.2026.1835509

**Published:** 2026-07-24

**Authors:** Samuel Adjorlolo, Lillian Akorfa Ohene, Florence Naab, Berit Mortensen

**Affiliations:** 1 Department of Mental Health Nursing, School of Nursing and Midwifery, University of Ghana, Accra, Ghana; 2 Department of Public Health Nursing, School of Nursing and Midwifery, University of Ghana, Accra, Ghana; 3 Department of Child and Maternal Health, School of Nursing and Midwifery, University of Ghana, Accra, Ghana; 4 Department of Nursing and Health Promotion, Oslo Metropolitan University, Oslo, Norway

**Keywords:** Ghana, midwives, needs assessment, nurses, perinatal mental health

## Abstract

**Background:**

Perinatal mental health (PMH) is a critical component of overall maternal well-being, yet the provision of PMH services remains challenging in Ghana and other settings. More crucially, there is limited data on healthcare providers' perspectives on PMH care delivery to inform efforts to improve service provision and mental health outcomes.

**Objective:**

This study explored healthcare providers' perspectives on current practices, gaps, and strategies to improve PMH care delivery in Ghana, a West African country.

**Methods:**

An exploratory qualitative design with a needs assessment orientation was adopted, and data were collected from focus group discussions with 32 nurses and midwives involved in perinatal healthcare service delivery in the Greater Accra Region of Ghana. Data were analysed using the six steps of reflexive thematic analysis.

**Findings:**

Four main themes were developed: Providers' Understandings of Perinatal Mental Health and its Intersection with Reproductive Health; Integrated Approaches to Perinatal Mental Health Care; Barriers and Challenges in Perinatal Mental Health Care; and Enhancing/Strengthening Perinatal Mental Health. Participants demonstrated knowledge of PMH and had adopted task-shifting and collaborative care approaches to provide PMH services. Key barriers to service provision include healthcare provider factors (e.g., stigma, inadequate training) and institutional challenges (lack of standardised tools, limited private consultation spaces, increased workload). Proposals to improve PMH outcomes include enhanced training, provision of resources and tools, and strengthened collaborative efforts.

**Conclusion:**

The findings highlight the need for comprehensive system reforms in PMH care delivery, including the provision of training and assessment tools and coordinated action across policy, institutional, and individual levels, supported by appropriate resource allocation and infrastructure development.

## 1 Introduction

Perinatal mental illness (PMI), defined as psychiatric disorders such as prenatal and postpartum depression and anxiety disorders occurring during pregnancy and about 1 year after delivery (1), represents a major public health threat globally (2). Approximately 11% of women experience perinatal depression, with those in low-resource settings reporting higher rates (13.1% vs. 11.4%) compared with their counterparts in high-income countries (3). African-specific data revealed a disturbing prevalence of prenatal depressive symptoms from 22.8% to 26.3% (4, 5). Relating to Ghana, the burden of antepartum depressive symptoms ranges from 19.7% to 50.5% (6–8), whereas postpartum depression was reported by 7% to 50.4% women (9, 10). Psychotic-like symptoms have also been documented among pregnant women in Ghana (11). PMI has been implicated in adverse birth experiences and outcomes such as prolonged labour, postpartum complications, preterm birth, low birth weight, and caesarean section (12–16). The foregoing provides enough impetus to invite healthcare practitioners to play an active role in preventing PMI and improving women's perinatal mental health (PMH).

Nurses and midwives constitute a critical mass of the healthcare workforce who are strategically positioned to identify and support women experiencing mental health challenges (17), as well as reduce or prevent factors that contribute to PMI, such as anemia (18). Nurses and midwives spend a considerable amount of time with pregnant and postpartum women, building rapport and trust that can be leveraged to inquire about and respond to PMH issues. Indeed, the Quality Maternal and Neonatal (QMNC) framework emphasizes a holistic approach to quality maternal care that also involves optimizing psychological processes to support woman's capabilities as part of the responsibilities of midwives and others working with pregnant and new mothers (19). This is particularly important given the reluctance of women in the perinatal period to initiate and seek help on their own volition (20, 21). In response, several countries, including Ghana, have instituted reforms and programs to promote access to mental health services, such as the creation of dedicated mental health units across the various levels of care (22). The QMNC framework recognizes the importance of sensitive and tailored maternal health service delivery that responds to the unique needs of childbearing women (19).

Some healthcare professionals have undergone Ghana Psychological Council certification programs in counseling to equip them with the requisite skills and knowledge to incorporate mental health services into primary, secondary, and tertiary healthcare delivery (23). These initiatives are envisaged to increase the availability and accessibility of mental health services across the various spectrum of healthcare. However, our understanding of the current practices, capabilities, and challenges faced by nurses and midwives in promoting PMH remains very poor. Recent studies have concluded that several challenges continue to beset PMH services in Ghana and beyond (24, 25), but how these challenges influence mental health service provision to women remains less explored. Furthermore, while global guidelines emphasize the integration of mental health screening and support into routine maternal health care (26), little is known about the various ways nurses and midwives have responded to this request. The current study expands the breadth of previous research by investigating existing practices, gaps, and strategies to promote PMH from the perspective of nurses and midwives in Ghana. Adopting an exploratory qualitative design with a needs assessment orientation, the study is analytically grounded in the QMNC framework, which positions psychosocial support, provider competence, and systems-level enablers as inseparable dimensions of quality maternal care. Consistent with the WHO health systems building blocks framework, which organises health system performance across the domains of service delivery, health workforce, information, medicines, financing, and governance, the study examines how these structural dimensions shape PMH care delivery at the frontline. The study is aimed at filling a critical void in the literature and, more crucially, to support the training, capacity-building, and policy development initiatives of nurses, midwives, and other healthcare professionals in PMH.

## 2 Method

### 2.1 Study design and setting

This is an exploratory qualitative study examining the perspectives of healthcare professionals providing care to women attending antenatal and postnatal clinics in Ghana, using focus group discussions (FGDs). The study adopted a needs assessment orientation, seeking to characterise existing practices, identify gaps, and generate evidence to inform workforce development and policy in PMH. The study was conducted as part of a broader project focusing on enhancing midwifery practice in Ghana through research, education and training. Consequently, the participants were recruited to explore their perspectives of sexual and reproductive health (SRH) service organisation and delivery in Ghana. Perinatal mental health was a pre-specified and structured component of the broader study, with dedicated sections of the interview guide addressing PMH definitions, service delivery approaches, barriers, and improvement strategies. The analysis reported here focuses exclusively on these PMH-relevant data segments, constituting a purposeful secondary thematic analysis of pre-defined thematic domains within the broader dataset. This approach is methodologically grounded in the literature on secondary qualitative analysis, which supports the rigorous re-analysis of data collected within one study for focused inquiry within a pre-specified thematic domain. The study was conducted in the Greater Accra Region (GAR), one of the 16 administrative regions of Ghana. The GAR is the most urbanised and densely populated region in the country, hosting the national capital, Accra, and a disproportionately high concentration of tertiary, secondary, and primary healthcare facilities relative to other regions. Maternal and reproductive health services in the GAR are therefore comparatively better resourced than in many other parts of the country, particularly the Northern, Upper East, and Upper West regions, where healthcare access is constrained by geographic remoteness, workforce shortages, and limited infrastructure. This geographic concentration means that the challenges to PMH service delivery identified in this study may represent a conservative estimate of the difficulties encountered nationally, underscoring the need for future research in rural and resource-constrained settings. The Ghanaian healthcare system is administratively structured as a three-tiered system (i.e., National, Regional, and District levels), but in terms of service delivery, the healthcare system is five-tiered: National, Regional, District, Sub-district and Community Health Planning and Services. Reproductive health services such as antenatal and family planning are provided at all levels of healthcare.

### 2.2 Participants and recruitment

The participants in this study were purposively sampled from healthcare facilities in the Greater Accra Region providing sexual and reproductive health (SRH) services. Eleven (11) facilities were purposively selected from a pool of institutions based on location (e.g., urban vs. peri-urban), and sociodemographic characteristics of service users, service type and geographical reach. A total of 32 participants were recruited from the 11 health facilities to participate in the study, averaging 3 participants per facility. To be included in the study, participants must have provided maternal healthcare to pregnant or postpartum women for at least two years prior to the commencement of the study. Other inclusion criteria included consent and willingness to participate in the study.

### 2.3 Data collection measures

The focus group discussion guide was developed as part of a larger study exploring the needs of healthcare professionals to provide sexual and reproductive health services in Ghana. The interview guide was pretested on six nurses and midwives from the University of Ghana School of Nursing and Midwifery. Revisions to the interview guide were made through consensus among the facilitators. The guide included four sections: The first section focused on the definition of SRH services, including mental health. The second section addressed the provision of SRH services. Questions included family planning, comprehensive abortion care, ante- and postnatal services, gender-based violence, the prevention and management of sexually transmitted infections and mental health services. The third section enquired about the challenges or barriers to the provision of these services, while the fourth section of the tool sought responses on what the participants needed to improve the provision of SRH services.

### 2.4 Data collection

Data were collected at the University of Ghana School of Nursing and Midwifery. Dedicated rooms were provided for each of focus groups. Consent was obtained at the time of recruitment and repeated on the day of data collection. Upon arrival at the venue for data collection, the participants were individually taken through the consenting process after the purpose of the study was explained to them and ample time was given for them to seek clarification. Thereafter, they were purposively assigned to five (5) groups to ensure diverse demographic backgrounds in each group. Each group consisted of five (5) to seven (7) participants. Group consent, in addition to individual consent, was sought by the facilitators, followed by the assignment of unique codes to each participant for anonymity, after which they provided background information on separate sheets. The codes were used to de-identify the participants to ensure confidentiality. The participants consented to audio recording the interactions purely for research purposes. Each FGD was facilitated by a trained researcher with experience in qualitative data collection, who adopted a non-directive stance, using open-ended prompts and follow-up probes to encourage free and candid expression from all participants. Icebreakers were provided by each facilitator to calm and relax the participants before the discussions. To ensure anonymity and enable transparent attribution of participant perspectives in the findings, each participant was assigned a unique alphanumeric code at the point of data collection. The letter in the code (A through E) denotes the focus group to which the participant belonged, corresponding to each of the five FGDs conducted, while the number identifies the individual participant within that group. For example, A2 refers to the second participant in FGD A, and E4 refers to the fourth participant in FGD E. These codes are used consistently throughout the Findings section to identify participant quotes. The study data, namely audio files and notes taken during the focus group discussions, were managed and stored using password protected computers. Data collection was done in English, which is the official language of communication in Ghana.

### 2.5 Data analysis

Data were transcribed by professional data transcribers with over five years of experience, using standardised procedures to ensure anonymisation of all transcripts. All transcriptions were reviewed by the facilitators of the focus group discussions. Consistent with the reflexive thematic analysis approach adopted, the study oriented towards thematic sufficiency rather than data saturation in the conventional sense. The analytical team reviewed transcripts iteratively across the five focus groups, and the recurrent appearance of themes and patterns across groups provided the basis for determining that sufficient data had been generated to develop a rich and coherent thematic account. Reflexive thematic analysis, using the six-steps, as outlined by Braun and Clarke (27) was adopted. The authors read the transcripts repeatedly to familiarize themselves with the content before coding. Through an inductive coding approach, a final codebook was generated. All project team members met to discuss the themes to ensure a reflexive process in the development and refinement of themes.

### 2.6 Ethics statement

Ethics approval was obtained from the Ghana Health Service Ethics Review Committee (GHS-ERC: 006/10/22). The study adhered to the principles outlined in the Declaration of Helsinki for human subjects' research. All participants were provided with detailed information about the study's purpose, procedures, and their rights. Written informed consent was obtained from each participant before data collection. Participation was voluntary, and participants could withdraw at any time without consequences. Confidentiality was maintained by using participant codes instead of names, and all data was securely stored with password protection.

### 2.7 Reflexivity and rigour

The study's trustworthiness was ensured through several strategies. Credibility was established through member checking, where selected participants were invited to review their interview transcripts and preliminary findings to confirm accuracy. Transferability was enhanced by providing detailed descriptions of the research context and participant characteristics. Dependability was maintained through an audit trail documenting all research decisions and procedures. Confirmability was achieved through researcher reflexivity, where researchers acknowledged and documented their potential roles throughout the research process. Additionally, investigator triangulation was employed, with multiple researchers independently analyzing the data before reaching consensus on themes. The COREQ (Consolidated Criteria for Reporting Qualitative Research) guidelines were followed to ensure comprehensive reporting of the study.

## 3 Findings

### 3.1 Sociodemographic characteristics of participants

The study included 32 participants, and their demographic characteristics are summarized in Table 1. The majority were females and those aged 36–45 years. Most participants specialised in midwifery. In terms of years of practice, the majority had 15 years or less of experience. Regarding rank or position, half of the participants were midwifery or nursing officer roles, followed by senior officers, and lastly, higher positions such as principal officers, deputy directors, or chiefs. Participants worked across various units, including maternity wards, public health, antenatal clinics, and other roles such as administration, research, and training.

**Table 1:** Demographic characteristics of participants.

Variable	Range/Category	Frequency	Percentage
Gender	Female	29	90.6
	Male	3	9.4
Age	20–35 years	10	31.2
	36–45 years	15	46.9
Specialty	46 + years	7	21.9
	Midwife	17	53.1
	Nurse	15	46.9
Years of practice	≤15 years	19	59.4
	>15 years	13	40.6
Rank/Position	Midwifery/Nursing officer	16	50
	Senior officers	11	34.4
	Principal officers/Deputy Director/Chief	5	15.6
Unit	Maternity ward	9	28.1
	Public Health	6	18.8
	Antenatal clinic	5	15.6
	Others	11	34.4

### 3.2 Findings from the reflexive thematic analysis

Four main themes were developed through the reflexive thematic analysis reflecting health care providers literacy, practices to optimize mental health, challenges and barriers and potentials for improvement. Within each main theme, necessary subthemes were added to describe differentiations within the themes, as can be seen in Table 2.

**Table 2:** Summary table of themes and subthemes.

Themes	Subthemes
Providers’ Understandings of Perinatal Mental Health and its Intersection with Reproductive Health	Defining Perinatal Mental Health
	Intersection of Mental, Sexual, and Reproductive Health
Integrated Approaches to Perinatal Mental Health Care	Task Shifting
	Collaborative Care
	Integrative Care
Barriers and Challenges in Perinatal Mental Health Care	Healthcare Provider-Based Factors
	Institution and Systemic Factors
Enhancing/Strengthening Perinatal Mental Health	Enhanced Training and Capacity Building
	Provision of Resources, Tools, and Manuals
	Policy and Institutional Support
	Collaborative Efforts and Holistic Care

#### 3.2.1 Theme 1. Providers' Understandings of Perinatal Mental Health and its Intersection with Reproductive Health

This theme explores participants' perceptions of maternal mental health and its connection with sexual and reproductive health. As stated by one of the participants: “…. *mental health in relation to our sexual and reproductive system is the woman having a state of comfort without questions in relation to their sexual organs, their reproductive health, when it comes to being intimate.” (B6).*

#### 3.2.2 Defining perinatal mental health

This subtheme centres on the meanings the participants ascribed to PMH. A notable trend across the interviews is the reference to “sound mind” or “state of mind” in the perinatal period.

This quote illustrates the view that a woman's state of mind speaks greatly about her mental health. The state of mind can be determined by the actions and inactions of women, defining their ways of life and their ability to be assertive and discerning. This empowers women to navigate the perinatal period confidently and well-equipped, helping to reduce unnecessary anxiety and foster better health outcomes. As noted by one of the participants: “*When an individual is well, especially women, and has the information they need, they become more discerning, more assertive, and they don't worry too much.”* (D2).

#### 3.2.3 Intersection of mental, sexual, and reproductive health

The subtheme highlights how the focus group discussed interconnectedness between mental health and reproductive health, emphasizing how these aspects influence each other. Participants expressed the belief that mental health challenges are not isolated but rather intertwined with sexual and reproductive health concerns.

“Mental health is a state of the mind that influences how we handle our reproductive decisions.” (B3)

“You need a sound mind to discern what's best for you regarding reproductive health.” (A2)

This intersection emphasizes the need for a holistic approach to care, recognizing how psychological well-being can influence reproductive choices, decisions, and outcomes. Participants emphasised that maintaining a balance between mental health and reproductive health is essential for achieving overall stability and well-being.

“If you are well in all aspects relating to your reproductive health, it also keeps your mind at ease.” (B6)

#### 3.2.4 Theme 2: integrated approaches to perinatal mental health care

This theme reflects the evolving approaches adopted to address maternal mental health within healthcare settings. Importantly, the practices described below were not uniformly present across all facilities or participants. Rather, they represent a spectrum of actual and aspirational practice: some participants — particularly those working in larger regional and district facilities with dedicated mental health units — described established, routine models of collaborative and integrative care, while others framed these approaches as desired but not yet fully implemented in their settings. This distinction is noted where relevant throughout the theme. Participants highlighted the significance of specific practices aimed at optimising care delivery through task shifting, collaborative efforts, and integrative strategies. As participant E4 described: “*So I escorted her to…. (the mental health)…. department, and then they had a thorough interaction, and then the client was helped. And she came back later and was so thankful that we were able to pick her on time and she's saved. So, I think when we have the department or the unit attached to our facilities, it helps.”.*

##### 3.2.4.1 Task shifting

Task shifting was described as a pragmatic response to the limited number of mental health professionals across the healthcare facilities. This practice was most vividly articulated by participants with longer clinical experience — particularly those with more than 15 years of practice — who described task shifting in detailed and pragmatic terms, drawing on accumulated clinical know-how. In this regard, the focus group discussions revealed how participants performed mental health service delivery roles beyond their professional training. This approach helped bridge service gaps and ensured basic mental health needs are addressed during antenatal and postnatal care, including case detection, counselling, psychoeducation, and referral, as highlighted by the following quotes, respectively.

“From the tone and what the client tells you, you can easily identify if the person is stressed or mentally unwell.” (E2)

“We take our time to explain to mothers how to handle stress and relax their minds. Sometimes we guide them through simple breathing exercises or help them think through their challenges to see things more positively.” (E3)

“We talk to them about their worries and encourage them, but serious cases are referred to counselors or specialists.” (E3)

##### 3.2.4.2 Collaborative care

Healthcare institutions with collaborative care partnerships are instrumental in addressing complex cases that go beyond the scope of general maternal health care. This integrated approach not only enhances the quality of care but also reinforces the need for interdisciplinary teamwork in promoting maternal well-being.

Collaborative care, as discussed by participants, underscores the value of establishing strong linkages between different healthcare units, ensuring that PMH is prioritised within a broader healthcare framework.

“We work hand in hand with the psychiatric team. While we address their maternal health needs, the specialists provide the counseling and therapy required for their mental well-being” (C4)

##### 3.2.4.3 Integrative care

The views of the participants reflect integrative care, where healthcare services are structured to ensure that maternal, mental, and public health needs are addressed seamlessly in a unified setting. This holistic approach empowers multiple healthcare professionals to work together within the same unit to address diverse needs and to ensure seamless care delivery, allowing mental health concerns to be addressed promptly by specialists like psychiatric nurses.

“In our facilities, we have integrated services so that at the reproductive and child health unit, you’ll find a midwife, a mental health nurse, and a public health nurse all working together. If a mental health issue arises, a psychiatric nurse is readily available to provide urgent care.” (B5)

##### 3.2.4.4 Theme 3: barriers and challenges

This theme captures the various obstacles faced by healthcare providers in addressing maternal mental health. These challenges span systemic, resource-related, cultural, and personal factors that impede the effective delivery of mental health services. These barriers were illustrated by one participant, who stated: “*if we were really equipped, we would be able to handle the medical and mental emergencies that happen on the ward. But we see ourselves calling the mental health unit because we have no idea what to even do for the client. So, I think basically most midwives should be taken through training on mental challenges for the client. The signs to look out for, what triggers it. because sometimes we trigger it. Yes, we on the labour ward. If we traumatize her during her labour and delivery….”* (D4).

##### 3.2.4.5 Healthcare provider-based factors

The participants discussed stigma and discrimination as healthcare provider-based factors that affect PMH service provision. The findings reinforce self-awareness among providers about the consequences of negative attitudes and behaviours, such as stigma and discrimination, that may alienate patients or prevent them from seeking help. As one participant suggested,

“…………then, stigmatization and discrimination on our part should be taken out of the equation,” (A2)

The participants also acknowledged that there was a significant gap in the training and preparation of healthcare providers to address maternal mental health. They highlighted the scant attention to mental health training as part of their professional training. Participants further emphasised that this deficit hinders their ability to engage effectively with patients facing psychological challenges, as they lack essential skills for basic counselling.

“There's a big knowledge deficit there… Even training on basic counseling is not mandatory for service providers.” (C5)

##### 3.2.4.6 Institution and systemic factors

Participants highlighted various institutional and systemic factors, including inadequate privacy in care settings, fragmented care, absence of screening tools, lack of protocols and manuals, and the burden of increased workload.

Regarding privacy in care settings, mothers appeared discouraged from openly discussing sensitive issues, including mental health concerns, since the presence of others in shared spaces creates a sense of vulnerability. This hinders case detection and management of mental health issues.

“The moment they see maybe another midwife palpating another woman, starts entering and going and coming, they just shun and they don't talk. But if it's one-on-one, the mothers are able to open up and you are likely to pick certain mental health issues.” (D1)

On mental health screening, the participants mentioned the absence of a standardised tool as a significant barrier. Without a simple and structured checklist, healthcare providers tend to struggle to detect mental health concerns, particularly in busy clinical settings or among staff with limited specialised training. This gap undermines the ability to provide timely interventions and contributes to inconsistencies in the quality of maternal mental health care.

“Currently, there is no tool or checklist for staff to quickly assess their clients, and this makes it difficult to identify mental health issues effectively.”(E5)

The increase workload narrative also reflected how heavy workloads among healthcare providers act as a barrier in an effort to promote PMH. Participants acknowledged that fatigue, time constraints, and competing demands in busy healthcare settings limit the opportunities to recognize subtle behavioral changes that might be indicative of PMH issues.

“Sometimes, because of our workload, we don't capture when the mental problem starts in a pregnant client.” (A4)

##### 3.2.4.7 Theme 4: enhancing/strengthening perinatal mental health

The participants reveal a pressing need to prioritize PMH within the healthcare systems through a multifaceted approach that addresses gaps in knowledge, resources, and system-wide support. The strategies proposed by the participants are outlined below.

##### 3.2.4.8 Enhanced training and capacity building in PMH

The participant highlights a gap in the curriculum for training nurses and midwives as it relates to PMH, including limited to no reference to PMH, and a short rotation in psychiatric units.

“During our training, mental health was barely touched—just one topic in the entire program. And even during rotations, we spent only two weeks in the psychiatry unit. If we want to take maternal mental health seriously, we need to start by building a solid foundation in the Nursing Training Colleges.” (C2)

Participants highlighted a gap in the practical skills and knowledge of nurses and midwives regarding PMH. Despite having mental health specialists within their facilities, they feel ill-equipped to identify women requiring specialised support. They also reported being inadequate to detect early signs and risk factors of PMH issues. They stressed the importance of equipping them with practical knowledge and skills through tailored training programs and continuous professional development.

“In my facility, we have a clinical psychologist and a psychiatrist, but we lack the skills to identify who needs help and when to refer them appropriately. I think we need more education and training through CPD courses.”(A5)

Another participant highlighted the importance of early intervention strategies advocated by others.

“Workshops and refresher courses on mental health would really help us identify and address these issues better.”(D2)

##### 3.2.4.9 Provision of resources, tools, and manuals

Participants emphasize the importance of structured and specialised guidelines for addressing PMH issues across antenatal, postnatal, and labour settings. They recognize the distinct mental health challenges that arise at different stages of the maternal journey and advocate for tailored approaches to ensure timely and appropriate care.

“Each stage—antenatal, postnatal, and labour—comes with its own challenges, and without clear guidelines, we can't address mental health effectively.” (C3).

“They should draw a protocol for us on mental health issues in relation to ANC—how to attend to our clients at ANC, labour ward, Obs & Gynae, theater, postnatal, everywhere.”(E4)

Additionally, the participants underscored the need for practical first-aid measures to address mental health emergencies within healthcare facilities. This perspective reflects a gap in current protocols and the urgency for immediate, standardised responses to crises, such as acute anxiety, postpartum psychosis, or severe depression, ensuring the safety and well-being of mothers in critical moments.

“We need immediate measures, like mental health first aid, to handle emergencies in the wards before they escalate.” (D3)

##### 3.2.4.10 Policy and institutional support

The participants emphasize the need for structured, policy-driven measures to enhance the delivery of PMH services. The request is to facilitate continuity of care to ensure trustful relationship between the healthcare providers and women. Participants highlight two critical aspects under this subtheme: counselling training and formalizing collabouration between mental health and maternal health services.

Participants advocate for making counseling training a mandatory component for healthcare professionals involved in maternal care. This they believe will ensure that healthcare providers are equipped with the skills to identify and address mental health issues in maternal settings effectively.

“I think they should make counseling training for nurses and midwives mandatory” (A2)

This quote reflects the call for policies that institutionalize the requirement for mental health training, ensuring consistent skill development among healthcare providers.

Participants stress the importance of formalizing collaborative practices between mental health and maternal health units to ensure seamless and integrated care. This could involve structured referral systems, shared protocols, and periodic assessments to align services.

“HeFRA (Health Facilities Regulatory Agency) should assess facilities periodically to ensure they provide comprehensive services.” (A4).

##### 3.2.4.11 Collaborative efforts and holistic care

Many participants underscored the value of multidisciplinary collabouration in addressing PMH. They advocated for a holistic approach that brings together healthcare providers, family and community support, and to strengthen the referral system.

Participants highlight the critical role of families and communities in providing emotional, psychological, and practical support for mothers. They advocate for family involvement in counseling and therapy to foster understanding and collective responsibility.

“I think it will be good to involve the family in counseling and therapy so that they can understand the mother's struggles better and know how to support her emotionally and practically.”(A5)

The participants emphasize the importance of efficient referral systems that will enable timely and appropriate care for mothers in need. This includes collabouration between the various levels of care and among different healthcare providers.

“Strengthening partnerships between midwives and psychiatric units key to facilitate referrals.” (E7)

This shared view of the participants underscores the need for structured pathways that link maternal health services with specialised mental health care.

## 4 Discussion

This study explored the perspectives of nurses and midwives on current practices, barriers, and strategies for improving PMH care delivery in Ghana. Analytically grounded in the Quality Maternal and Neonatal Care (QMNC) framework and the WHO health systems building blocks, the findings illuminate how structural, institutional, and cultural forces converge to shape PMH service delivery at the frontline. The results carry important implications for the design and implementation of interventions to improve PMH in Ghana and across the sub-Saharan African region. The QMNC framework emphasises the importance of psychosocial support as an integral part of empowering women in the perinatal period (19). The new mother's wellbeing is essential for her own life as well as the health of the newborn and the whole family [WHO (26)]. The study has provided important insights into the strengths and persistent gaps of PMH initiatives in Ghana. The acknowledgement of mental health as a crucial component of perinatal health and a key resource for reproductive health decision-making is central to promoting PMH. This aligns with the World Health Organisation's guidance on integrating mental health into maternal healthcare (26), reflecting the growing recognition that PMH is inseparable from other aspects of wellbeing (28, 29). Indeed, the recognition that mental health challenges are intertwined with sexual and reproductive health concerns reinforces existing research (30, 31). This understanding has significant implications for service delivery, and may explain the motivations behind task shifting, collaborative and integrated healthcare practices reported by the participants. Critically, however, task shifting must not be understood merely as a pragmatic workforce solution. Viewed through a power relations lens, it also represents a structural repositioning of complex mental health responsibilities onto the least specialised cadre of providers — nurses and midwives — without commensurate training, formal authority, or institutional recognition. This underscores the importance of accompanying task-shifting policies with robust capacity-building, standardised protocols, and sustainable supervision structures, rather than treating expanded provider roles as a default response to specialist shortages. These approaches align with global trends in addressing mental health workforce shortages, particularly in resource-limited settings (32, 33). The World Health Organisation's endorsement of task shifting as an effective strategy is supported by numerous studies that demonstrate positive outcomes in various contexts (26). Studies by Simas, Flynn (34) have demonstrated that integrated care models lead to better outcomes and increased access to services. The emphasis on interdisciplinary teamwork and the integration of services within reproductive and child health units aligns with recommendations from systematic reviews by Engelhard, Hishinuma (35), which highlight the effectiveness of unified service delivery models.

The foregoing notwithstanding, the study revealed several crucial barriers and challenges, stemming from multiple sources, which undermine the proposals by the QMNC and WHO for providing psychosocial services to women in the perinatal period. Viewed critically, these barriers are not merely operational inconveniences; they reflect deeper structural and cultural dynamics that sustain the marginalisation of mental health within maternal care systems. Healthcare provider-based barriers, particularly stigma and discrimination, were clearly critical impediments to PMH initiatives, an observation that is consistent with previous research (36, 37). Importantly, provider stigma in this context cannot be understood as merely an individual attitudinal failure. It is reproduced within professional cultures shaped by the longstanding biomedical dominance of maternal care, in which psychological symptoms are frequently secondary to physical monitoring and clinical tasks. The limited formal training in mental health communication compounds this, leaving providers without the vocabulary, confidence, or institutional mandate to engage with mental health disclosures sensitively. Reducing provider stigma therefore requires systemic intervention — including curriculum reform, supervised reflective practice, and institutional leadership that models mental health as a core clinical priority — rather than relying on individual awareness-raising alone. The identified gap in healthcare provider training and preparation mirrors findings from global studies on PMH care delivery (38, 39), highlighting a persistent challenge in healthcare workforce development. Institutional and systemic challenges were recorded. Concerns about lack of privacy in care settings were frequently presented as a significant barrier that threatens patients' willingness to disclose mental health concerns and seek support (40). The absence of standardised assessment tools and protocols, coupled with increased workload, creates additional barriers to effective care delivery. The absence of private consultation spaces deserves particular interpretive attention: it is not simply a logistical oversight but a tangible expression of how institutional spaces are organised around the priorities of physical over psychological care. When women cannot disclose mental health concerns in private, the burden of identification falls entirely on providers who must navigate clinical conversations in shared, semi-public ward environments — an arrangement that structurally privileges physical examination over psychological inquiry. This is consistent with evidence from the Ghanaian context, where Kumador et al. (2025) found that institutional resources — including access to qualified professionals, standardised information, and a supportive care environment — were decisive mediators of maternal mental health trajectories for mothers of preterm infants at Korle Bu Teaching Hospital. Together, these findings underscore how the organisation of institutional space and resource allocation reflect implicit priorities that must be challenged through purposeful policy. These findings parallel those of Vogel, Moore (41), who documented how resource constraints and standardization gaps impact maternal mental health care quality across various healthcare settings.

The significant nature of the existing challenges, consistent with previous findings in Ghana (42), calls for swift interventions. As healthcare practitioners who are familiar with the healthcare system, the participants offered some useful strategies to promote PMH by addressing the challenges above. The emphasis on enhanced training and capacity building supports previous research by Smith (43), and has implications for care quality and consistency (41). Healthcare systems should implement standardised screening tools and protocols, such as the Edinburgh Postnatal Depression Scale (44), while addressing workload through improved staffing allocation (42). The call for practical skills development and continuous professional education aligns with recommendations from international and Ghanaian-based studies on healthcare workforce development (17, 42, 45, 46). The identified need for resources, tools, and manuals highlights a critical gap in current maternal healthcare systems. This finding reinforces previous research by Costa, Santos (47) on the importance of standardised guidelines and protocols in improving maternal mental health care quality. The participants’ emphasis on stage-specific guidelines for antenatal, postnatal, and labour settings reflects growing recognition of the unique mental health challenges at different stages of the maternal journey.

Policy and institutional support were obviously crucial requested for improving maternal mental healthcare. The recommendation for mandatory counseling training aligns with global best practices in maternal healthcare workforce development (48). The emphasis on formalizing collabouration between mental health and maternal health services supports previous findings on the effectiveness of integrated care models (28, 49). The results of this study have substantial implications for healthcare practice across various levels of service delivery. System-wide reforms are necessary to develop and implement policies that effectively combine mental and reproductive health services, while ensuring adequate resources are allocated for training programs and supporting healthcare workers in expanded roles.

### 4.1 Implications and recommendations

The findings of this study carry crucial implications for healthcare practice and policy across multiple system levels. The recommendations below are organised into short-term actionable measures and long-term systemic reforms, each explicitly grounded in the study's findings. In the short term, healthcare institutions should prioritise the creation of private consultation spaces, a finding consistently raised as a barrier to disclosure across all five focus groups, as a relatively low-cost structural change that would immediately improve the conditions for mental health identification and support. Concurrently, facilities should implement validated standardised screening tools, such as the Edinburgh Postnatal Depression Scale (44), to address the critical absence of structured assessment protocols identified by participants. Targeted continuing professional development workshops on PMH, encompassing case identification, basic counselling skills, and referral pathways, should be introduced promptly, given participants' express recognition that their training had not equipped them for mental health practice. These short-term measures are actionable at the facility and district levels and do not require significant legislative change. In the longer term, systemic reforms are necessary to achieve sustainable improvements. These proposals will require purposeful structural change within healthcare organisations and the broader policy environment. Policies supporting integrated mental and reproductive health services are essential, alongside adequate resource allocation for training and task-shifting initiatives. Nursing and midwifery education programmes require substantive revision to incorporate comprehensive PMH content and embedded psychiatric rotations, a recommendation directly supported by participants' reports of having received minimal mental health training during their pre-service education. This effort should be led by the Nursing and Midwifery Council, supported by the Ministry of Health and development partners. Additionally, formalising collaborative care arrangements between maternal and mental health units, including through HEFRA periodic facility assessments to verify compliance, would institutionalise the interdisciplinary practices that currently depend on individual initiative. Heavy workload, identified as a systemic barrier, should be addressed through evidence-based staffing allocation policies that recognise the expanded PMH responsibilities of nurses and midwives. These long-term reforms, when implemented, must be accompanied by appropriate resource allocation to ensure effective and sustainable improvements in PMH care delivery across all levels of the health system.

### 4.2 Future research directions

Looking forward, these findings suggest the need for a fundamental shift in maternal healthcare delivery towards more integrated, collaborative approaches, given that they have proven to be effective (50). It is recommended that future research should focus on evaluating the effectiveness of these integrated approaches in the Ghanaian context and identifying implementation best practices. As suggested by recent studies (51), particular attention should be paid to the sustainability of task-shifting initiatives and the development of culturally appropriate intervention models that addresses psychosocial issues in perinatal women within the QMNC framework. Future studies should take into consideration the rural vs. urban divide and investigate collaborative care practices. Currently, there is limited data regarding the provision of PHM services at healthcare institutions located in deprived and resource-constrained rural geographies, as well as the challenges affecting their efforts. Such insights would support resource distribution and allocation to ensure universal access to PMH care services. Longitudinal studies examining the impact of enhanced provider training and standardised protocols on maternal mental health outcomes would be particularly valuable. Furthermore, research investigating the role of family and community support in PMH care could provide insights into developing more comprehensive care models in the Ghanaian context. These research directions would help build an evidence base for effective implementation strategies and inform ongoing policy development in PMH care in Ghana.

### 4.3 Strength and limitations

The strength of this study is the engagement of nurses and midwives from diverse professional backgrounds and varied experiences based on years of practice and different levels of healthcare delivery (e.g., District, Municipal, Regional and security hospitals, polyclinics and health centres), providing a rich and multifaceted account of PMH service delivery in the Greater Accra Region. The participants were purposively selected to obtain information-rich data, and the use of multiple rigour strategies — including member checking, investigator triangulation, and an audit trail — strengthens confidence in the credibility of the findings. Nevertheless, several limitations must be acknowledged. The study was conducted exclusively in the Greater Accra Region, which is the most urbanised and best-resourced region in Ghana. This geographic concentration introduces an urban bias, and the findings may not fully reflect the experiences of nurses and midwives working in rural, remote, or resource-constrained settings where PMH service challenges are likely to be substantially more acute. The use of focus group discussions, while appropriate for capturing shared professional experiences, may have introduced social desirability bias, particularly on sensitive topics such as provider stigma. The group setting may have discouraged some participants from expressing dissenting or self-critical views, despite the mitigating strategies employed (including diverse group composition, anonymous participant codes, and non-directive facilitation). Future research should employ individual interviews to complement FGD findings on sensitive topics. Additionally, the overrepresentation of female participants reflects the general nursing and midwifery workforce composition in Ghana, but may limit the perspectives captured on gender dynamics in PMH service delivery. Despite these limitations, the consistency of findings with prior research in the Ghanaian and broader LMIC context supports the quality of the data generated and the relevance of the study.

## 5 Conclusion

This study makes three distinct contributions to knowledge on PMH service delivery. First, it provides one of the most systematic qualitative examinations of PMH service delivery from the perspective of frontline nurses and midwives in Ghana — a population whose voices are underrepresented relative to those of patients or specialist clinicians in the existing literature. Second, it documents an organic, though unevenly distributed, adoption of task-shifting, collaborative care, and integrative care models by Ghanaian nurses and midwives in the absence of formal policy mandates — a finding that is novel in the Ghanaian context and resonant with broader LMIC literature on workforce adaptation under resource constraints. Third, and in contrast to many HIC-based studies that locate barriers primarily at the individual provider level, this study reveals that the most consequential barriers in the Ghanaian context are institutional and systemic — including the absence of standardised tools, inadequate training infrastructure, and the organisation of clinical space that privileges physical over psychological care — pointing to the need for structural rather than purely individual-level interventions. While barriers include provider stigma, inadequate training, and institutional challenges such as lack of privacy and resources, promising practices including task shifting and collaborative care were identified. These practices, however, require formal policy support, adequate resourcing, and accompanying capacity-building to move from individual initiative to sustained system-wide practice. The findings collectively emphasise the need for coordinated reforms across policy, institutional, and individual levels — reforms grounded not in aspiration alone, but in the lived experiences and practical wisdom of the nurses and midwives who navigate this work every day.

## Data Availability

The datasets presented in this study can be found in online repositories. The names of the repository/repositories and accession number(s) can be found in the article/Supplementary Material.
